# The effectiveness of the mental health social worker‐led multiprofessional program in preventing long‐term hospitalization and readmission in acute psychiatric inpatients in Japan: A retrospective analysis

**DOI:** 10.1002/pcn5.192

**Published:** 2024-04-14

**Authors:** Yuta Yanagisawa, Hiroshi Kimura, Hideki Komatsu, Hiroyuki Watanabe, Masaomi Iyo

**Affiliations:** ^1^ Department of Psychiatry Chiba University Graduate School of Medicine Chiba Japan; ^2^ Department of Psychiatry Gakuji‐kai Kimura Hospital Chiba Japan; ^3^ Department of Psychiatry International University of Health and Welfare Narita Japan; ^4^ Division of Medical Treatment and Rehabilitation Chiba University Center for Forensic Mental Health Chiba Japan

**Keywords:** long‐term hospitalization, mental health social worker, new long‐stay, readmission

## Abstract

**Aim:**

The large number of new long‐stay (NLS) patients and high readmission rates in psychiatric hospitals are longstanding concerns in Japan despite reforms to encourage multidisciplinary support of such patients. Staffing shortages of specialists, especially mental health social workers (MHSWs), may be one of the reasons for these problems to remain unsolved.

**Methods:**

The authors examined the effectiveness of the MHSW‐centered multidisciplinary care model in preventing NLSs and rehospitalization in terms of both patient dynamics and cost by retrospective comparison of before and after program implementation.

**Results:**

After our program was introduced, NLS was almost completely prevented. In addition, a significant decrease in readmissions of involuntarily admitted patients was also observed. On the other hand, the resulting decrease in treatment costs and hospital revenues was mismatched by an increase in personnel costs.

**Conclusion:**

While MHSW‐centered multidisciplinary care is effective for the community integration of patients, there are cost challenges. State policy changes are needed to resolve staffing problems, along with the introduction of appropriate indicators of community integration.

## Graphical abstract

The large number of new long‐stay (NLS) patients and high readmission rates in psychiatric hospitals are longstanding concerns in Japan. The authors examined the effectiveness of the mental health social worker (MHSW)‐centered multidisciplinary care model in preventing NLSs and rehospitalization in terms of both patient dynamics and cost by retrospective comparison of before and after program implementation. While MHSW‐centered multidisciplinary care is effective for the community integration of patients, there are cost challenges.

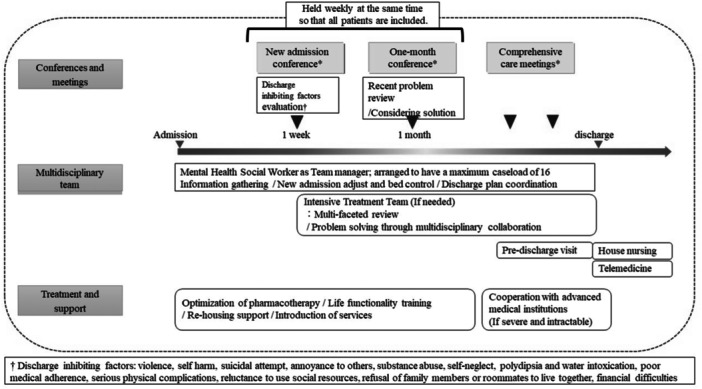

## BACKGROUND

A longstanding problem in Japanese psychiatric care has been the large number and long duration of inpatient admissions. In 2003, the number of psychiatric beds per 1000 population in Japan was 2.78, by far the highest among Organization for Economic Cooperation and Development countries.^1^ In 2021, the number was still 2.57. Between 2003 and 2013, the total number of new long‐stay (NLS) patients reaching a 1‐year mark after admission remained almost unchanged at approximately 50,000.^2^ According to a 2016 survey, the discharge rate 1 year after admission was 85.7%.^3^ Taken together, the situation has remained largely unchanged for the past 20 years.

Another problem is the high readmission rate. In a 2017 survey, as many as 17% and 35% of patients were readmitted to a psychiatric ward within 3 months and 1 year of discharge, respectively.^4^ These rates are limited to patients discharged from psychiatric hospitals within a year, and the rates for long‐stay patients are even higher. These data suggest a “revolving door” situation in this field.

Several studies in other countries have suggested that behavioral and environmental problems are important causes of long stays and rehospitalizations of psychiatric patients,^5^, ^6^, ^7^, ^8^, ^9^, ^10^, ^11^, ^12^, ^13^ and the same is likely true in Japan. Questionnaire surveys conducted by nurses working in psychiatric wards in Japan showed that problems in self‐care and interpersonal relationships are vital factors contributing to prolonged hospitalization.^14^, ^15^ In Japan, a survey of factors preventing psychiatrists from discharging long‐stay patients within 1 year revealed that behavioral and social problems such as poor adjustment within the family, difficulty securing a place to live after discharge, problematic behavior, and a decline in activities of daily living (ADLs) or instrumental activities of daily living (IADLs) were causes in 17%–51% of such cases.^16^ Only 33% of patients with severe mental illness discharged from psychiatric wards use community services after discharge,^17^ indicating that inadequate access to social resources to support their lives in communities (LICs) contributes to their rehospitalization.

Reforms to encourage multidisciplinary support in psychiatric hospitals, with the aim of improving these issues, were instituted. As a main example of this effort, the Mental Health and Welfare Act was partially amended in 2014, adding the requirement of assigning a counselor to supervise the postdischarge living arrangements (PDLAs) of patients admitted for medical care and protection. It was envisaged that mental health social workers (MHSWs), who are professionals in mental health welfare in Japan, would take on this counselor role to address the behavioral and social problems of the patients.^18^, ^19^

Unfortunately, these reforms did not change the abovementioned hospitalization trends. One of the reasons appears to be a staffing issue, especially for MHSWs. The law stipulates that the maximum number of patients assigned to a counselor is 50, but surveys indicate that the actual number of assigned patients ranges from 16 to 33,^20^ due mostly to the extensive, long‐term involvement required for each patient. This may have caused a discrepancy between basic labor costs and actual payments. The same research revealed that approximately 20% of counselors assigned to PDLAs were actually non‐MHSW professionals,^19^ possibly indicating a shortage of MHSWs in the hospitals.

**Figure 1:**
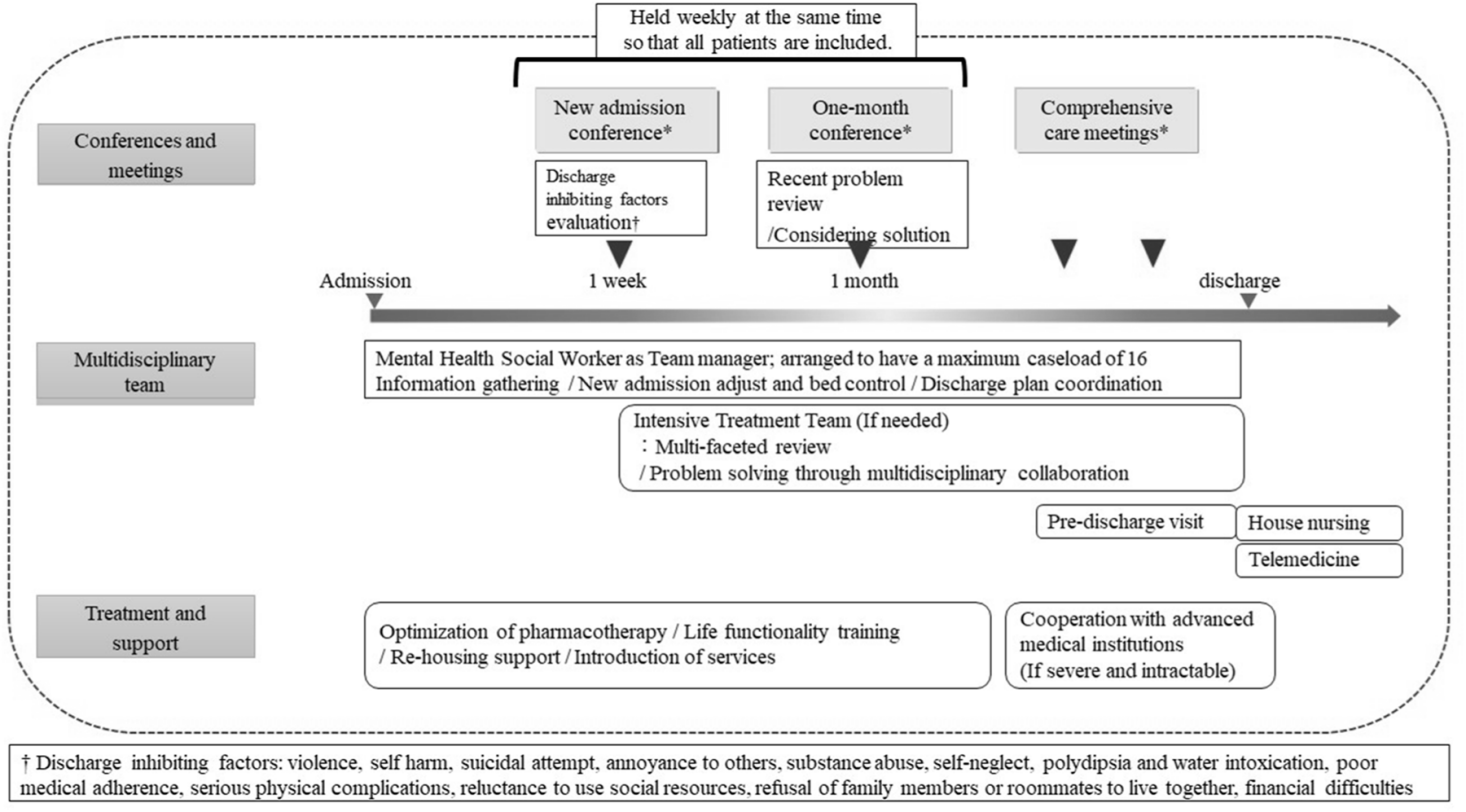
The path of the new long‐stay prevention program (see also Supporting Information). The program has the following key features: management of the progress of the treatment by mental health social workers, identification of factors interfering with discharge, a new admissions conference procedure, a 1‐month conference with multidisciplinary participation from doctors, nurses, mental health social workers, occupational therapists, psychotherapists, day care staff, and local community supporters, formation of an individual treatment team (ITT) if needed, and further comprehensive care meetings.

Data are lacking regarding the effectiveness of MHSW‐based support in the real world, especially in terms of cost and hospital management. Thus, the authors sought to examine the effectiveness of MHSW‐centered multidisciplinary support in terms of both patient dynamics and cost. The NLS prevention program, a multidisciplinary support program to prevent NLSs and rehospitalizations, was introduced during Fiscal Year (FY) 2016 at the inpatient unit of a single‐specialty psychiatric hospital in Chiba, Japan. The program defined the treatment structure and process shown in Figure 1 (also see Supporting Information). The method of multidisciplinary intervention of course depends on the patient's disease and condition. In general, prior to the introduction of this program, information sharing was mainly done through medical records, and multidisciplinary meetings were held only occasionally; in addition, interventions by MHSWs, occupational therapy, and psychologists were only implemented when proposed by the physician. No upper limit was set for the number of cases each MHSW undertook. On the other hand, after the program was introduced, a new admission conference within a week of admission and a discharge support conference every month became mandatory, and conferences with family members and community assistance providers were actively held under the leadership of MHSWs. These conferences are used to coordinate professional support. Predischarge visits are also proactively conducted in coordination with MSHWs. Staffing on the wards is optimized so that the maximum caseload of each MHSW is about 16.

With the launch of this program, the authors instituted a retrospective cohort study to examine its effects. The primary objective of this study was to investigate whether this program could prevent NLSs and rehospitalizations after discharge. The authors also assessed the cost and hospital management outcomes for inpatient and outpatient treatment in the year following hospitalization.

## METHODS

As a preliminary note, the authors experienced several changes in hospital management and staffing during the period in which the program was implemented. Starting in FY2017, the hospitalization categories for care of inpatients on the ward changed from an “acute care unit” to “emergency psychiatric.” Both hospitalization categories have provisions such as a discharge rate within 3 months for the calculation, but reimbursement and staffing are more extensive for the emergency psychiatric category. Because of this change, some personnel were transferred to the emergency ward from other wards. Three MHSWs were dedicated to 48 beds in the emergency ward, fulfilling the provisions of the NLS program. Consequently, the number of beds was reduced while the number of personnel remained the same.

Clinical and cost outcomes were compared between patients admitted before and after the program implementation. FY2014 and FY2015 were designated as the non‐program period and FY2017 and FY2018 as the program period, with the transition period of FY2016 for program implementation being excluded from either period. After excluding hospitalizations for forensic psychiatric evaluation and the second or subsequent hospitalizations for patients who were hospitalized multiple times, hospitalizations during FY2014–2015 and FY2017–2018 were assigned to the non‐program and program groups, respectively.

Data related to hospitalizations of both groups were extracted from electronic medical charts and collected in a dataset for statistical analysis. Using registered data from the psychiatry emergency unit, variables included age, gender, diagnosis, length of stay (LOS), whether or not the admission occurred via the emergency psychiatry information center (which coordinates out‐of‐hours referrals), and the modalities of admission (voluntary or involuntary). Outpatient visits and readmissions during the year after discharge were also investigated. The rehospitalization rates were analyzed for patients who (1) were discharged to home or a welfare facility from the eligible hospitalization and (2) were traceable 1 year after discharge, that is, they continued to attend the outpatient clinic at the hospital or were in our hospital at the 1‐year anniversary of discharge.

Treatment cost changes and hospital management indicators were also investigated. Treatment costs were researched for up to 1 year after hospitalization in both inpatient and outpatient contexts by selecting one of every five cases in order of their admission. Patients who were not traceable 1 year post admission were excluded from the calculation. To address the changes in our hospital's inpatient ward charge category, the inpatient hospitalization costs of the non‐program group were recalculated, converting from acute care unit charges to emergency psychiatric charges. The daily hospitalization charges at that time were as follows: in the emergency psychiatric category 35,570 yen up to 30 days after admission and 31,250 yen at 31 days and thereafter; in the acute‐care‐unit category, 19,840 yen up to 30 days and 16,550 yen at 31 days thereafter. Our program required an additional 15,730 yen up to 30 days and 14,700 yen at 31 days and thereafter; these amounts were added to the non‐program group inpatient medical care costs.

This study was approved by the ethics committee of the Chiba University Hospital (No. 3176) and the ethics committee of the Gakuji‐kai Kimura Hospital (No. 2018‐03)

## STATISTICS

All calculations and regression analyses were performed using SPSS version 27.0 (SPSS Inc.). Analysis of readmission risk was carried out using Kaplan–Meier survival curves with readmission to the hospital as event occurrence and the log‐rank test. Continuous and categorical variables were compared by independent *t*‐test and the Fisher's exact test, respectively. All calculations were provided with confidence intervals and used P‐values for significance testing. The significance level was set at 5% bilaterally.

## RESULTS

A total of 892 hospitalizations were included in the study as eligible cases. There were no significant differences in mean age, percentage of patients with each diagnosis, or mean LOS between the two groups (P > 0.05, Table 1). There were significant increases in the percentages of involuntary admissions and admissions via the emergency psychiatry information center in the program group (P < 0.001 for both items, Table 2).

**Table 1:** Patients’ characteristics in both groups.

		Non‐program	Program	
	*n*	429	460	
Age at hospitalization				
Mean	Year (SD)	48.2 (17.0)	49.3 (16.1)	NS
≥65	*n* (%)	85 (19.8)	91 (19.8)	NS
≤18	*n* (%)	10 (2.3)	11 (2.4)	NS
Gender, male	*n* (%)	185 (43.1)	180 (39.1)	NS
Main diagnostic (ICD‐10)	*n* (%)			NS
F0		8 (1.9)	17 (3.7)	
F1		14 (3.3)	14 (3.0)	
F2		272 (63.4)	282 (61.3)	
F31		36 (8.4)	51 (11.1)	
F3 (except F31)		61 (14.2)	67 (14.6)	
F4		23 (5.4)	13 (2.8)	
F8		2 (0.5)	5 (1.1)	
Others		13 (3.0)	11 (2.4)	

Abbreviations: ICD‐10, International Classification of Diseases 10th Revision; NS, not significant; SD, standard deviation.

**Table 2:** Admission status and length of stay.

		Non‐program	Program	
	*n*	429	460	
Admission status				
Via emergency psychiatry information center	*n* (%)	33 (7.7)	105 (22.8)	P < 0.001
Involuntary admission	*n* (%)	249 (58.0)	346 (75.2)	P < 0.001
Length of stay, mean	Days (SD)	62.6 (97.1)	61.3 (83.4)	NS

Abbreviations: NS, not significant; SD, standard deviation.

**Figure 2:**
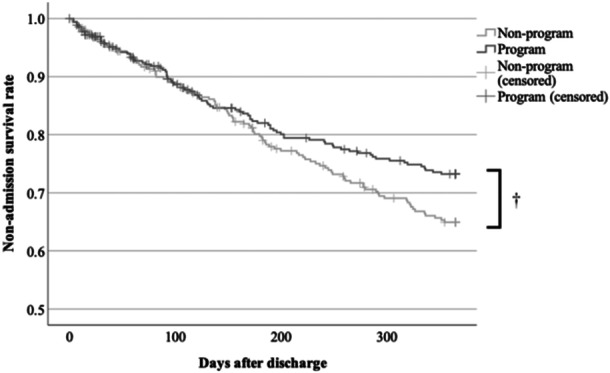
Non‐admission survival rate after discharge (total eligible admissions, ^†^P > 0.05).

**Figure 3:**
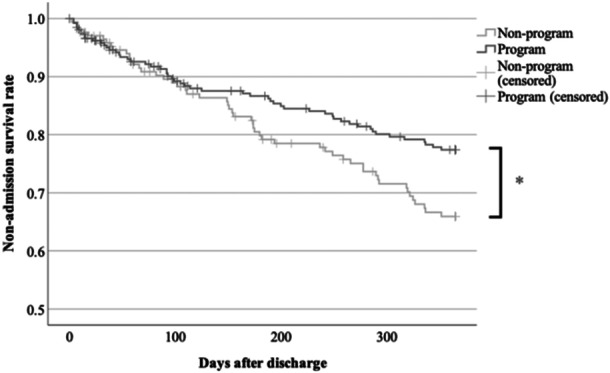
Non‐admission survival rate after discharge (involuntary admission, *P < 0.05).

In 2018, 1 year after the program was introduced, the 1‐year discharge rate reached 100%, demonstrating complete suppression of NLSs (Table 3). The readmission rate and survival curve the year after discharge are shown in Table 4 and Figure 2. Especially for patients admitted involuntarily, readmissions were significantly decreased in the program group (P < 0.05, Figure 3).

**Table 3:** Discharge rate 3/12 months after admission.

		Non‐program		Program	
	FY	2014	2015	2017	2018
	*n*	222	209	257	204
3 months after admission	*n* (%)	208 (93.7)	199 (95.2)	250 (97.3)	197 (96.6)
1 year after admission	*n* (%)	220 (99.1)	206 (98.6)	253 (98.4)	204 (100)

Abbreviation: FY, fiscal year.

**Table 4:** Readmission rate.

		Non‐program	Program	
Total eligible admission	*n*	311	358	
Traceable after discharge				
3 months	*n* (%)	287 (92.3)	311 (86.9)	P < 0.05
1 year	*n* (%)	270 (86.8)	307 (85.8)	NS
Readmission (among traceable)				
3 months	*n* (%)	31 (10.8)	30 (9.3)	NS
1 year	*n* (%)	99 (36.7)	86 (28.0)	P < 0.05
Involuntary admission	*n*	172	266	
Traceable after discharge				
3 months	*n* (%)	156 (90.7)	240 (90.2)	NS
1 year	*n* (%)	146 (84.9)	226 (85.0)	NS
Readmission (among traceable)				
3 months	*n* (%)	17 (10.9)	22 (9.2)	NS
1 year	*n* (%)	52 (35.6)	54 (23.9)	P < 0.05

Abbreviation: NS, not significant.

**Figure 4:**
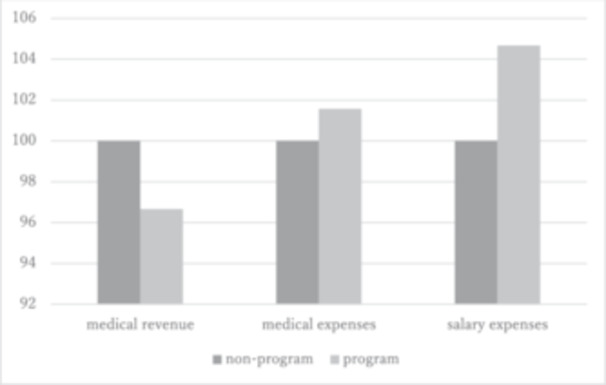
Change in medical revenue and expenses (non‐program as 100%).

As for the economic aspect, a decrease in treatment costs and hospital revenues was observed, along with an increase in personnel costs (Table 5 and Figure 4). The 50 patients selected from either of the groups for cost analysis were not significantly different in terms of the percentages of involuntary admissions and admissions via emergency psychiatry, which might have influenced the readmission rate. The increase in personnel costs was associated with the staffing changes mentioned above.

**Table 5:** Change in medical costs.

		2015	2018	
Total number of administrations the costs of which were calculated	*n*	50	50	
Admission modality at hospitalization, voluntary	*n* (%)	23 (46.0)	14 (28.0)	NS
Medical costs				
Total	Yen	3,425,172	3,320,441	NS
Inpatient	Yen	3,224,511^a^	2,983,715	NS
Outpatient	Yen	200,661	336,726	NS

Abbreviation: NS, not significant.

^a^ Charge converted from acute care unit hospitalization charges to emergency psychiatric hospitalization charges.

## DISCUSSION

NLS suppression and reduction in the rehospitalization rate of patients admitted involuntarily were observed in the program group. These changes were accompanied by an increase in the number of involuntary admissions and admissions through the emergency psychiatry information center. As patients who were admitted involuntarily or as an emergence may have required more urgent attention and experience, these results would indicate that our program contributed to the prevention of NLSs and rehospitalizations. In other words, it can be expected that MHSW management played a role in promoting a smoother transition and integration into LIC from psychiatric hospitalization.

Given that our hospital's NLS incidence was originally relatively low compared to all of Japan, the more meaningful result is the rate of prevented readmissions. There has been a change in the indicators emphasized in terms of national policy as well. A Vision for Reform of the Mental Health Care System, which was set out by Japan's Ministry of Health, Labour and Welfare (MHLW) in 2004, initially specified only the rate of patients remaining in the hospital for 1 year or less and the discharge rate of patients who had been in the hospital for 1 year or longer as target indicators.^21^ However, in 2017, MHLW launched a new philosophy of building a community‐based integrated care system for people with mental illness (the integrated care system) and announced the introduction of new indicators, such as readmission rates, in its medical and disability welfare plans.^22^

For involuntarily admitted patients, the program was significantly effective in reducing readmission rates. Consistent with the data in this study, involuntarily hospitalized patients currently account for the majority of users of emergency psychiatric care in Japan, therefore the effectiveness of this program can be evaluated as being in line with the needs of the field.

Conversely, the program did not significantly reduce readmissions for the entire population, including voluntary admissions. Several previous studies in other countries have shown that readmission is high among patients admitted voluntarily.^23^, ^24^ One possible explanation for this is that patients who are able to self‐manage their symptoms to some extent may be using voluntary hospitalization as a respite. A retrospective study conducted in Denmark—a country that has reduced its number of psychiatric ward beds through deinstitutionalization efforts over the past 50 years—observed that the average LOS for patients with schizophrenia had decreased, although the readmission rate within 1 year of discharge had increased from 50% to 70%.^25^ This result could mean that deinstitutionalization may cause a revolving door situation if treatment during hospitalization or LIC support is insufficient; the converse view is that hospitalization provides a means for patients to protect themselves from crisis or that patient exacerbations are addressed earlier in the community.

Therefore, readmission rates may not truly reflect the effectiveness of the prevention of symptom exacerbation and community integration for persons with mental disorders. Thus, the policy has been reviewed with the idea that there may be a more appropriate indicator than the readmission rate. In the mid‐term review of Japan's medical plan in 2020, the number of community life days (CLDs) per year after discharge was proposed as a new indicator to replace the readmission rate.^26^ With the introduction of this indicator, if readmissions do occur but hospital stays are kept short with appropriate interventions, the CLD values will increase and the outcomes of intervention will be more properly evaluated. The effectiveness of our program may also be more appropriately evaluated using the CLD evaluation system.

In terms of costs and hospital management, both the benefits and the challenges are apparent from our data. Regarding the economic burden for the general society, the annual medical costs per patient have decreased. Although the outpatient medical costs grew, the decrease in the inpatient medical costs more than made up for this increase. The reduction in NLS and readmissions after the implementation of the program may have contributed to this outcome. Such an analysis of combined inpatient and outpatient costs is important for evaluating the integrated care system and should be continued in the future.

In contrast to the actual medical costs, this result of a decline in revenues clarifies an important problem for individual hospitals. Our hospital's finances worsened after the program was implemented because salary expenses increased despite decreases in hospitalization charge revenues. This was entirely due to the staffing changes required to place more MHSWs into emergency wards.

The placement of sufficient numbers of MHSWs may be the most essential element for this program to continue to be effective, and the managerial feasibility of sufficient MHSW placement will determine the versatility of this program. The number of patients a single counselor for PDLAs can reasonably be responsible for is more limited than previously estimated, as the report presented above demonstrates. In psychiatric emergency medicine, the counsellor is required to coordinate highly individualized elements such as housing, employment, schooling, symptom management, and community support systems to ensure a smooth transition and settlement of patients into LIC. The individuals needing to be involved in this process include not only the patients themselves, but also the patients’ families, community support workers, and government officials. Most importantly, since discharge within 3 months is required in emergency or acute wards, the counselor is required to work quickly and continuously from the moment of the patient's admission to the point of discharge. Without assurances that individual hospitals have the management foundation to hire enough people to make such quick and sufficient intervention possible, the program cannot thrive.

Although the number of MHSWs per capita in Japan is not small compared to other high‐income countries, the excess number of beds has led to relative understaffing.^27^, ^28^ Policy changes for correcting the imbalance in the number of hospital beds and reimbursement for MHSW staffing will be necessary for MHSW‐led multidisciplinary programs such as our program to be effective.

Several limitations must be considered in the interpretation of our findings. First, some variation in intervention may exist. In some of our cases, treatment during the first 3 months to 1 year after admission is not carried out in the emergency wards where the NLS prevention program is implemented, meaning that the effectiveness of the program may not be fully reflected. This is due to an essential contradiction in the Japanese psychiatric system between the LOS allowed in the emergency ward, that is, 3 months, and the LOS that the NLS implies, that is, 1 year. In addition, our ward experienced several changes in staffing and ward operations owing to the change in the admission category. These changes may have affected the LOS or readmission rate independent of the implementation of the program.

Second, some background data for patients and treatment environments are missing or differ between the two groups. Only medical records were used, and symptom severity was not measured with an evaluation scale. Unevaluated differences in severity may have impacted the discharge and readmission rates.^10^ However, there was no significant difference in the diagnoses between the two inpatient groups, and since the number of admissions via the emergency psychiatry information center and the number of involuntary admissions were both increased in the program group (FY2017–2018), it is not likely that the severity in the program group was lower compared with that of the non‐program (FY2014–2015) group. The routes to admission and the admission type may in themselves affect treatment outcomes but were not controllable in the analysis because this research was a naturalistic cohort study.

Third, although data on the role of each professional are important for this program to be adapted to each hospital's actual situation and applied widely, there is a lack of data on inpatient and outpatient psychiatric treatment. Regarding medication, clinically diagnoses in our study were varied, and the sample size of each group divided by mental disorder was too small to be evaluated for medication. In our study, no data are available for number of occupational therapy sessions and type of occupational therapy. As individual occupational therapy has been reported to have reduced readmissions,^29^ interventions by such professionals may have a significant role in promoting LIC per se.

Finally, treatment outcomes are not adequately assessed. This includes changes in medication, psychiatric symptoms, cognitive function, social functioning, motivation, and treatment satisfaction. The actual living conditions of people with mental disabilities, including their employment, schooling, and use of supports, must be taken into account when considering the quality of community life and its improvement cost.

In the integrated care system, medical care is considered only part of a comprehensive community life that includes disability welfare and care, housing, social participation (employment), community support, and education. In the future, it will be important to conduct comprehensive evaluations that include indicators related to the actual living conditions to build this system.

## CONCLUSION

The multidisciplinary support program with MHSWs as team leaders was proven to effectively prevent NLSs and rehospitalizations, and reduce medical costs, including those of outpatient treatments in hospitals. However, for this program to be implemented widely, hospital staffing and management issues must be resolved. To realize policy changes that will address this challenge, further research is needed that, for instance, calculates CLDs and analyses actual living conditions in the community and their associated costs.

## AUTHOR CONTRIBUTIONS

Hiroshi Kimura, Hiroyuki Watanabe, and Masaomi Iyo conceived and designed this study. Yuta Yanagisawa, Hiroshi Kimura, Hideki Komatsu, and Hiroyuki Watanabe collected and collated the data. Yuta Yanagisawa and Hiroshi Kimura wrote the main manuscript text, prepared the tables and figures, collected and collated the data, and analyzed the data. All authors read and approved the final manuscript.

## CONFLICT OF INTERESTS STATEMENT

Dr. Kimura has received speaker's honoraria from Janssen Pharmaceutical K.K., Meiji Seika Pharma Co. Ltd., MSD K.K., Otsuka Pharmaceutical Co. Ltd., Sumitomo Pharma Co. Ltd., Takeda Pharmaceutical Co. Ltd., and Teijin Pharma Co. Ltd., and a Grant‐in‐Aid for Young Scientists (B) from the Japan Society for the Promotion of Science (20K16640). Dr Iyo is a consultant of Otsuka Pharmaceutical Co. Ltd. and Sumitomo Pharma Co. Ltd. Dr Iyo has received speaker's honoraria from Janssen Pharmaceutical K.K., Otsuka Pharmaceutical Co. Ltd., and Sumitomo Pharma Co. Ltd. Dr. Masaomi Iyo is an Editorial Board member of *Psychiatry and Clinical Neurosciences Reports* and a co‐author of this article. To minimize bias, they were excluded from all editorial decision‐making related to the acceptance of this article for publication. The remaining authors declare no conflict of interest.

## ETHICS APPROVAL STATEMENT

All methods of this study were performed in accordance with the Declaration of Helsinki. All experimental protocols of this study were approved by the ethics committees of the Chiba University Hospital (No. 3176) and Gakuji‐kai Kimura Hospital (No. 2018‐03).

## PATIENT CONSENT STATEMENT

In accordance with the approval of the Ethics Committee mentioned above, notification of the study was added to the hospital's homepage or notice board, and patients and their family members were allowed to opt out of the study.

## CLINICAL TRIAL REGISTRATION

N/A.

## Supporting information

Supporting information.

## Data Availability

The data that support the findings of this study are available from the Gakuji‐kai Kimura Hospital, although restrictions apply in regard to their availability. They were used under license for the current study and so are not publicly available. Data are available from the authors upon reasonable request and with permission of the Gakuji‐kai Kimura Hospital.
