# Supplementary material for: The effectiveness of the mental health social worker‐led multiprofessional program in preventing long‐term hospitalization and readmission in acute psychiatric inpatients in Japan: A retrospective analysis
Source: PCN Rep. 2024 Apr 14;3(2):e192. doi: 10.1002/pcn5.192 (PMC11114310; doi:10.1002/pcn5.192)
Supplement: Supplementary file 1 — Supporting information. [file PCN5-3-e192-s001.docx]

Supplementary materials 1 NLS prevention program

We drafted “the comprehensive New Long Stay prevention program” to promote discharge of inpatients within less than one year from acute psychiatric wards. The program consists of the methods shown in fig. 1. These methods aim at smooth and prompt provision of housing and livelihood support, and improvement of severe and intractable psychiatric symptoms, which are the main reason of long hospitalization.

Because in acute psychiatric wards in Japan the human resources of specialists are scarce, and there are demands to discharge people in a limited period, multidisciplinary treatment with high cohesion and smooth management are essential method. In order to achieve this, this program assigns MHSWs the role of managing the progress of the treatment by gathering information, adjusting acceptance of new admission and bed control, and coordinating a plan to discharge.

Within a week after admission, the presence of the discharge inhibiting factors is identified by the attending doctor of the hospitalization. These factors were selected on the basis of “START”^[[1]](#footnote-1)^. The multidisciplinary treatment and support will be provided targeting the factors identified here.

In order to share and concretely examine the personal discharge inhibiting factors, a new admission conference is held once a week with the participation of doctors, nurses, MHSWs, occupation therapists, psychologists, day care staffs, local community supporters, and other officials inside and outside the hospital. If the problems that lead to prolonged hospitalization are significant, varied, or complex, we organize an individual treatment team (ITT) composed of multidisciplinary specialists to conduct a multifaceted review of the problems, identify issues that require intervention, and resolve them through collaboration of the specialists. The management of ITT is also a role of MHSWs.

All inpatients who have been hospitalized for four weeks are on the agenda for a one-month conference, which held on the same schedule as the new admission conference. At the conference, the recent problems are reviewed, risk for prolonged hospitalization is assessed and solutions are considered by the participating multidisciplinary specialists.

Details of problems which cannot be addressed in the whole staff conference are discussed in a comprehensive care meeting for each patient, involving the patient, family, and outside stakeholders. Throughout the hospitalization period, there will be a total of 3-5 opportunities for information sharing and discussion of treatment and support policies at the in-hospital conferences and comprehensive care meetings. Comprehensive care meetings might be held outside the hospital.

Optimization of pharmacotherapy is one of the major issues in inpatient treatment, along with multidisciplinary life functionality training, re-housing support, and introduction of services to support daily living. Efficacy and tolerability of medications are evaluated based on the interviews and observations of doctors, nurses, occupational therapists, and other multidisciplinary professionals. Since the introduction rate of long-acting injectable antipsychotics (LAIs) in Japan is lower compared to other countries^[[2]](#footnote-2)^, LAIs are actively considered when indicated, taking into account the patient's wishes, physical condition, and adherence to medication.

If the illness is severe and intractable, highly specialized treatments will be considered in close cooperation with advanced medical institutions such as university hospitals.

In order to prevent re-admission, effective measures must be taken during hospitalization. Introduction of daily living support and home nursing are the frequently applied measures, yet they are inadequate for some patients who fail to receive outpatient care or whose symptoms worsen too quickly. For early detection and early response to the signs of relapse in such patients, telemedicine will be actively considered in this program.

We introduced this program as a pilot program in a psychiatric emergency ward of Gakujikai Kimura Hospital (Kimura Hospital) from February 2017. After the introduction, we will periodically check whether the program is operating as planned.

1. START: The Short-Term Assessment of Risk and Treatability. O'Shea LE, Picchioni MM, Dickens GL. [The Predictive Validity of the Short-Term Assessment of Risk and Treatability (START) for Multiple Adverse Outcomes in a Secure Psychiatric Inpatient Setting.](https://pubmed.ncbi.nlm.nih.gov/25724192/) Assessment. 2016 Apr;23(2):150-62. doi: 10.1177/1073191115573301. Epub 2015 Feb 27. [↑](#footnote-ref-1)
2. [↑](#footnote-ref-2)
